# The Impact of the Aryl Hydrocarbon Receptor on Antenatal Chemical Exposure-Induced Cardiovascular–Kidney–Metabolic Programming

**DOI:** 10.3390/ijms25094599

**Published:** 2024-04-23

**Authors:** You-Lin Tain, Chien-Ning Hsu

**Affiliations:** 1 Division of Pediatric Nephrology, Kaohsiung Chang Gung Memorial Hospital, Kaohsiung 833, Taiwan; tainyl@cgmh.org.tw; 2 Institute for Translational Research in Biomedicine, Kaohsiung Chang Gung Memorial Hospital, Kaohsiung 833, Taiwan; 3 College of Medicine, Chang Gung University, Taoyuan 333, Taiwan; 4 Department of Pharmacy, Kaohsiung Chang Gung Memorial Hospital, Kaohsiung 833, Taiwan; 5 School of Pharmacy, Kaohsiung Medical University, Kaohsiung 807, Taiwan

**Keywords:** cardiovascular disease, dioxins, metabolic syndrome, chemical, chronic kidney disease, aryl hydrocarbon receptor, developmental origins of health and disease (DOHaD), prenatal exposure, hypertension

## Abstract

Early life exposure lays the groundwork for the risk of developing cardiovascular–kidney–metabolic (CKM) syndrome in adulthood. Various environmental chemicals to which pregnant mothers are commonly exposed can disrupt fetal programming, leading to a wide range of CKM phenotypes. The aryl hydrocarbon receptor (AHR) has a key role as a ligand-activated transcription factor in sensing these environmental chemicals. Activating AHR through exposure to environmental chemicals has been documented for its adverse impacts on cardiovascular diseases, hypertension, diabetes, obesity, kidney disease, and non-alcoholic fatty liver disease, as evidenced by both epidemiological and animal studies. In this review, we compile current human evidence and findings from animal models that support the connection between antenatal chemical exposures and CKM programming, focusing particularly on AHR signaling. Additionally, we explore potential AHR modulators aimed at preventing CKM syndrome. As the pioneering review to present evidence advocating for the avoidance of toxic chemical exposure during pregnancy and deepening our understanding of AHR signaling, this has the potential to mitigate the global burden of CKM syndrome in the future.

## 1. Introduction

Numerous epidemiological and experimental findings have established that early life exposure to adverse environmental conditions can significantly impact the likelihood of developing adult-onset diseases [1,2]. This phenomenon, now recognized as developmental programming or the developmental origins of health and disease (DOHaD), elucidates the adaptations made by a developing fetus in response to cues during early life. These adaptations result in morphological and functional adjustments that may prove detrimental in later life stages, thereby increasing the susceptibility to adult diseases.

A myriad of early life factors can trigger developmental programming, including maternal malnutrition, maternal illnesses, complications during pregnancy, substance abuse, medication usage, or exposure to chemicals during pregnancy [1,2,3,4,5,6]. Environmental chemicals possess the ability to traverse the placental barrier, and it is widely acknowledged that the prenatal stage is particularly vulnerable to chemical disruptions and subsequent health ramifications compared to later developmental stages [7]. Concurrent exposure to multiple chemicals can exacerbate health consequences either through additive or synergistic effects [8]. Notably, the scale of chemical production has expanded significantly over the last six decades [9], with nearly 8000 chemicals now being manufactured or imported in substantial quantities.

Cardiovascular–kidney–metabolic (CKM) syndrome has surfaced as a significant and pressing global public health issue [10]. The impact of CKM syndrome is thought to affect approximately 40% of the adult population in the United States [10]. In 2023, the American Heart Association initially recognized CKM syndrome as a systemic ailment defined by complex physiological interconnections among metabolic disorders, chronic kidney disease (CKD), and cardiovascular health [11]. The likelihood of adverse consequences is heightened as a result of the interplay of these factors, leading to multiorgan dysfunction [11]. CKM syndrome is categorized into four discernible stages, ranging from stage 0 to stage 4, each representing a different degree of advancement and severity across the intricate spectrum of this condition. Across the complex range of CKM syndrome, different pivotal components emerge at various stages, contributing to the nuanced evolution and severity observed. Of paramount importance to emphasize is that prioritizing early prevention, rather than solely focusing on treatment, holds the potential to improve the global burdens associated with CKM syndrome.

The DOHaD theory establishes a connection between early life programming and various recognized facets of CKM syndrome, including cardiovascular disease (CVD) [6], metabolic disease [12], hypertension [13], CKD [14], and obesity [15]. Several molecular mechanisms linked to CKM programming have been discovered, such as an aberrant renin–angiotensin system (RAS), epigenetic dysregulation, deficient nitric oxide (NO), disturbances in nutrient-sensing signals, oxidative stress, and gut microbiota dysbiosis [16,17,18,19,20,21]. Conversely, by targeting these pivotal mechanisms, there is a shift in focus from managing diseases during adulthood to intervening in disease processes before they clinically manifest, known as reprogramming, which holds promising potential as a preventive strategy.

The aryl hydrocarbon receptor (AHR) is a pivotal ligand-activated transcription factor recognized for its capacity to sense environmental chemicals and regulate various physiological processes, including fetal development [22,23,24]. Extensive research over the years has elucidated how activation of AHR by environmental chemicals or microbial-derived uremic toxins impacts the cardiovascular, renal, and metabolic systems, thus contributing to the development of different facets of CKM syndrome [25,26,27].

Although the detrimental effects of AHR activation due to adult exposure to environmental chemicals on cardiovascular–kidney–metabolic health are well-established, our understanding of its involvement in the chemical-induced programming of CKM syndrome remains limited. Therefore, this review aims to delineate the impact of prenatal chemical exposures on CKM programming by synthesizing the available epidemiological and experimental evidence, with a particular emphasis on AHR signaling. Furthermore, we discuss potential interventions targeting AHR for reprogramming purposes to mitigate the onset of CKM syndrome.

A comprehensive search of scientific databases, including MEDLINE, SCOPUS, Embase, and the Cochrane Library, was conducted to elucidate the intricate relationship between AHR, CKM syndrome, and developmental programming. This exploration encompassed various keywords and their permutations, including “cardiovascular disease”, “chronic kidney disease”, “obesity”, “fatty liver”, “metabolic syndrome”, “diabetes”, “hypertension”, “hyperlipidemia”, “pregnancy”, “gestation”, “lactation”, “progeny”, “offspring”, “mother”, “developmental programming”, “DOHaD”, “reprogramming”, “aryl hydrocarbon receptor”, “endocrine-disrupting chemicals”, “organophosphate flame retardants”, “phthalates”, “microplastics”, “heavy metals”, “air pollution”, and “PM_2.5_”. Additionally, supplementary investigations were selected and evaluated based on relevant references identified in eligible papers. The final search was concluded on 20 March 2024.

## 2. Aryl Hydrocarbon Receptor

### 2.1. The Structure of AHR

**Figure 1:**
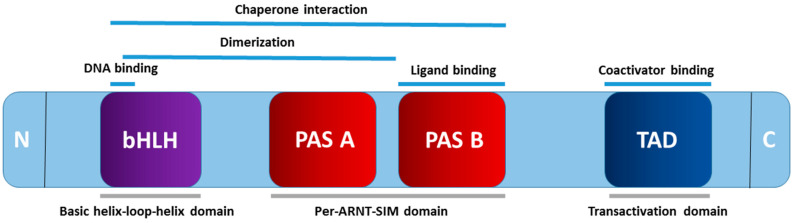
Schema outlining the structure of the aryl hydrocarbon receptor.

Belonging to the basic helix–loop–helix Per–ARNT–SIM (bHLH–PAS) family is the AHR, with its structure comprising an N-terminal bHLH domain, a central PAS domain (A and B), and a C-terminal transactivation domain [22] (Figure 1). The N-terminal bHLH domain of AHR undergoes dimerization, resulting in the formation of a four-helical bundle. This configuration serves as the DNA-binding domain and facilitates dimerization [28]. Governing DNA recognition, ligand binding, and chaperone interactions are the roles of the PAS domain [29]. Additionally, this domain, in conjunction with the bHLH domain, aids in mediating the heterodimerization of AHR with the aryl hydrocarbon receptor nuclear translocator (ARNT) [30]. Diverse ligands are accommodated by the ligand-binding domain (LBD) situated within the PAS B domain [31]. Within the C-terminal transactivation domain (TAD), the Q-rich subdomain takes precedence in the transcriptional activation of xenobiotic response elements (XRE) in the DNA [32]. Moreover, binding to coactivators during transcription exhibits a broad spectrum of diversity and tissue-specific effects [33].

### 2.2. AHR Ligands

Environmental pollutants, as well as dietary- and microbiota-derived metabolites, are the major sources of AHR ligands. These ligands can be delineated as either exogenous or endogenous. The former category includes dietary compounds like polyphenols, environmental contaminants such as dioxins, pharmaceuticals like omeprazole, and a variety of synthetic compounds like SP600125. Endogenous AHR ligands include compounds synthesized within the human body (e.g., tryptamine) and those generated by the gut microbiota (e.g., indoles). The activation of AHR by these distinct classes of ligands precipitates context-dependent positive and negative consequences.

### 2.3. AHR Signaling

**Figure 2:**
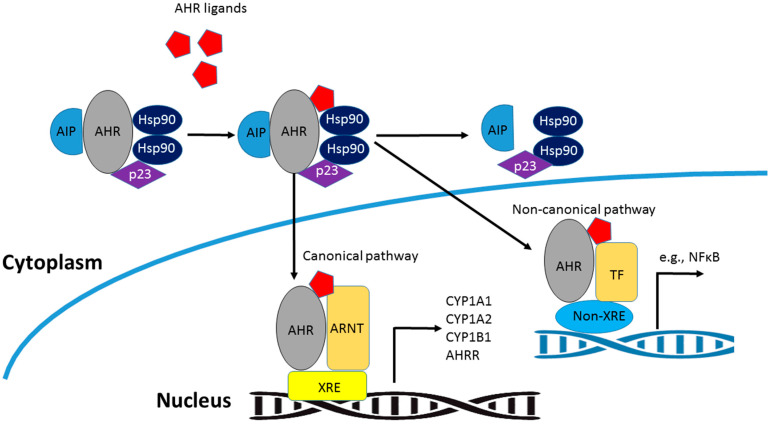
Classical and non-classical AHR signaling pathways.

The activation of AHR and its subsequent downstream signaling transduction comprises both canonical and non-canonical pathways [34] (Figure 2). Within the canonical pathway, AHR forms complexes with molecular chaperones, remaining inert in the cytosol [35]. Included among these molecular chaperones are heat shock protein 90 (Hsp90), AHR-interacting protein (AIP), also known as ARA9 and XAP2, and p23 [36]. Upon binding with a ligand, AHR undergoes a structural alteration, leading to the release of the AHR/ligand complex from the chaperone proteins. Following this, AHR translocates to the nucleus, where it forms a heterodimer with ARNT. Subsequently, this AHR/ARNT complex binds to xenobiotic response elements (XREs) located within the regulatory regions of target genes, thus modulating their expression. Noteworthy AHR target genes encompass those encoding members of the cytochrome P450 superfamily enzymes (such as CYP1A1, CYP1A2, and CYP1B1), as well as the AHR repressor (AHRR), which have a critical role in detoxifying environmental chemicals and negatively regulating AHR-dependent gene expression, respectively [37].

In the non-canonical signaling pathway, once AHR is activated, it interacts with different transcription factors inside the nucleus, thereby facilitating its attachment to non-XRE DNA elements, consequently regulating the expression of target genes [38]. For instance, the interaction of AHR binding to transcription factors (e.g., NFκB) triggers specific downstream gene expression [39]. In light of the fact that the promoters of most AHR-regulated genes do not contain identifiable XREs [40], there is speculation that AHR could potentially cooperate with a multitude of other nuclear factors to control gene expression following exposure to diverse exogenous and endogenous ligands.

## 3. AHR and CKM Syndrome

AHR elicits varied physiological effects contingent upon its localization within distinct tissues. AhR is expressed ubiquitously, while its distribution changes significantly with age [41]. During fetal development, AhR demonstrates distinct distribution in the liver, kidneys, lungs, pancreas, thymus glands, testicles, and epithelial cells, with fairly diminished levels observed in the heart, aorta, and brain. In adulthood, AHR exhibits elevated expression in the placenta, lungs, spleen, pancreas, and liver, while displaying comparatively reduced abundance in the brain, heart, and skeletal muscles [42]. For a number of years, AHR was primarily investigated for its involvement in organ toxicity induced by environmental chemicals, given that many of these chemicals contain ligands for AHR. Increasing evidence suggests that AHR is involved in triggering pathogenesis in various components of CKM syndrome [25,26,27].

### 3.1. Cardiovascular Disease and Hypertension

Despite its low expression in the fetal heart, the AHR signaling pathway is critical for cardiac development. Genetic deficiency in AHR is associated with cardiac hypertrophy and developmental vascular defects in the heart, kidney, and liver [43,44]. Additionally, the process of cardiomyocyte differentiation is meticulously controlled by AHR signaling. Activation, inhibition, or suppression of AHR can all potentially impact the differentiation of cardiomyocytes derived from mouse embryonic stem cells [45].

AHR is also involved in the regulation of the vascular microenvironment [46]. Ischemia-induced angiogenesis was noticeably augmented in AHR knockout (KO) mice compared with that in wild-type animals, which was associated with enhanced ARNT [47]. Another study revealed that ischemic insult increases AHR expression and its transcriptional activity in neurons in vitro and in vivo, while ablation of AHR by pharmacological or genetic loss-of-function approaches leads to neuroprotection [48]. In addition, acute kynurenine administration, an AHR ligand, causes vascular dysfunction accompanied by oxidative stress [49]. Moreover, exposure to environmental chemicals containing ligands of AHR (e.g., dioxins, PAH, and benzo[a]pyrene) is reported to promote the development and progression of atherosclerosis [25].

Although the exact mechanism has yet to be fully determined, AHR is involved in the complex networks that control blood pressure (BP). AHR KO mice developed hypotension at low altitudes and hypertension at modest altitudes, which might be related to elevated plasma endothelin-1 levels [50]. In the administration of captopril (an angiotensin-converting enzyme (ACE) inhibitor) to heterozygous and homozygous AHR KO mice, it was observed that the heterozygous group exhibited a significantly greater reduction in blood pressure compared to the homozygous group [51], accompanied by higher plasma renin and ACE activity in the heterozygote AHR KO mice. These findings suggest the interplay between AHR and vasoconstrictors in the regulation of BP.

### 3.2. Kidney Disease

In CKD, the buildup of uremic toxins within the body poses significant risks to all tissues and organs. Among these toxins, the AHR plays a pivotal role, acting as a receptor for many uremic toxins [26]. Notably, tryptophan-derived uremic toxins such as indoxyl sulfate and indole acetic acid are known AHR ligands, contributing to kidney inflammation and the progression of CKD [26].

AHR signaling holds crucial importance in maintaining the delicate balance between regulatory T (Treg) cells and T helper type 17 (Th17) cells in CKD. However, this pathway can be dysregulated by environmental chemicals, leading to aberrant activation [52]. Depending on the specific ligand and cellular context, AHR activation can either exacerbate or mitigate inflammation. Aberrant activation of AHR signaling may trigger inflammation by promoting monocyte adhesion, increasing the expression of pro-inflammatory cytokines, and reducing the bioavailability of nitric oxide (NO) [53,54]. Conversely, AHR can also exert anti-inflammatory effects. Additionally, AHR interacts with other pathways such as Nrf2, peroxisome proliferator-activated receptor-γ (PPAR-γ), and NF-κB, contributing to the diverse responses of AHR during different stages of CKD progression [55].

Moreover, AHR competes with hypoxia-inducible factor 1-alpha (HIF-1α) in binding to ARNT, influencing pro-inflammatory responses [56]. Furthermore, AHR antagonizes transforming growth factor beta 1 (TGF-β1) signaling in fibrogenesis, suggesting the potential of targeting AHR to attenuate CKD progression [57].

### 3.3. Diabetes, Obesity, and NAFLD

AHR KO mice displayed low plasma insulin, imbalanced glucose homeostasis, and impaired glucose intolerance [58], indicating the significance of AHR expression in the regulation of glucose balance.

The involvement of AHR/CYP1A1 activation is suggested in the development of non-alcoholic fatty liver disease (NAFLD) and the consequent onset of diabetes [59]. AHR activation leads to decreased levels of PPARα, consequently impacting β-oxidation. This reduction is associated with diminished expression of PEPCK and G6Pase, both recognized for their roles in controlling hyperglycemia and insulin resistance [27]. Additionally, given the circadian variation in PPARα, it modulates the levels of CLOCK and BMAL1, thereby impacting glucose tolerance and disturbing the regulation of specific metabolic genes [60].

AHR plays a role in regulating adipocyte differentiation by modulating the PPAR signaling pathway, which is essential for regulating fatty acid oxidation and glucose metabolism [61]. Dioxins like TCDD bind to AHR, triggering inflammation in adipocytes and consequently leading to impairment in insulin sensitivity. Additionally, the assembly of AHR–ARNT complexes interferes with several signaling pathways. The activation of AHR by TCDD additionally disrupts lipoprotein lipase activity in adipose tissue, thus regulating adipocyte differentiation and interfering with the PPAR signaling pathway crucial for fatty acid oxidation and glucose metabolism [62]. Furthermore, the upregulation in TNF-α expression induced by TCDD exacerbates dysfunction in insulin signaling and insulin resistance.

## 4. Epidemiological Evidence: The Link between Chemical Exposure and CKM Syndrome

Presented in Table 1 are the principal sources and documented detrimental impacts associated with CKM syndrome in human research, attributable to various environmental chemicals encountered during routine consumer activities. Numerous adverse effects on cardiovascular–kidney–metabolic health are posed by a plethora of environmental chemicals. Subsequent sections will delve into a discussion of each of these chemicals.

### 4.1. Dioxins

Dioxin, the most extensively researched and toxic variant, is formally known as 2,3,7,8-tetrachlorodibenzo-pdioxin (TCDD). “Dioxins” typically refers to a group of closely related chemical compounds, including polychlorinated dibenzo-p-dioxins (PCDDs), dioxin-like polychlorinated biphenyls (PCBs), and polychlorinated dibenzofurans (PCDFs), which share similar chemical structures and properties. Dioxins are predominantly released from anthropogenic activities such as pesticide manufacturing, wood pulp bleaching, and waste incineration [90]. Accumulating in the food chain within the environment and persisting for extended periods in the body’s fat tissue [91], dioxins can be encountered by pregnant mothers through the consumption of diets high in animal fat or via occupational exposure. Elevated exposure to dioxins has been linked to several facets of CKM syndrome, including cardiovascular disease [63], diabetes [64], metabolic syndrome [65], kidney disease [66,67], and hypertension [67].

### 4.2. Plastic Chemicals

The proliferation of plastic waste presents a significant environmental predicament, with a substantial portion of plastic being non-recyclable. Consequently, it infiltrates our surroundings, polluting oceans and disrupting ecosystems. Comprising a carbon backbone and augmented with numerous additional chemicals to form polymers, plastics harbor a plethora of toxic compounds including neurotoxicants, carcinogens, and endocrine disruptors such as Bisphenol A (BPA), di-2-ethylhexyl phthalate (DEHP)—the most prevalent phthalate, and organophosphate flame retardants (OPFRs). Furthermore, as plastics degrade, they fragment into microplastics (0.1–0.5 mm in diameter) and nanoplastics (1–1000 nm in diameter), exacerbating their detrimental impact on human health.

BPA has a characteristic structure that mimics estrogens by binding to their receptors [92], which causes it to be classified as an endocrine-disrupting chemical (EDC). Human exposure to BPA occurs primarily via the hydrolysis of polycarbonate plastics utilized in food and liquid containers, and medical devices. Several recent epidemiological studies suggest that BPA exposure is connected to the risk of developing cardiovascular disease [68], diabetes [69], obesity [69], NAFLD [70], hypertension, and kidney disease [66,67]. Importantly, evidence from mother–child cohort studies revealed that childhood obesity [93] and hypertension [94] could be related to maternal exposure to BPA.

People are constantly exposed to phthalates via plastic containers, food packaging, and medical devices. Similar to BPA, phthalates are recognized as EDCs and have been linked to all facets of CKM syndrome [66,67,71,72,73,74]. Another notable plastic compound is organophosphate flame retardant (OPFR), predominantly utilized as flame retardant plasticizers in engineering plastics [95]. OPFRs have been detected in indoor environments and are extensively employed in consumer goods like plastics, rubbers, construction materials, and electronic devices [95]. Emerging research has underscored the connections between OPFR metabolites and various components associated with CKM syndrome in humans, as outlined in Table 1 [75,76,77,78].

Microplastics (MPs) resulting from the environmental degradation of plastic waste are pervasive across diverse ecosystems, although the precise health risks to humans remain indeterminate [96]. Human exposure to MPs can range between 74,000 and 121,000 particles annually [96]. Accumulation of MPs has been observed in human blood, feces, breast milk, and certain organs [97]. A recent investigation involving 304 patients with carotid artery disease revealed that those with detectable MPs within atheroma were at heightened risk for cardiovascular events compared to those without such detection [79]. Nonetheless, there remains a dearth of information regarding the potential associations between MP exposure and other components of CKM syndrome.

### 4.3. Per- and Polyfluoroalkyl Substances (PFAS)

Per- and polyfluoroalkyl substances (PFAS) constitute a class of chemicals utilized in the production of fluoropolymer coatings and products engineered to resist water, heat, oil, and grease [98]. Epidemiological investigations have unveiled correlations between exposure to certain PFAS and a spectrum of CKM manifestations, encompassing obesity [80], diabetes [80], NAFLD [80], hypertension [67], and kidney disorders [99]. A longitudinal study on birth cohorts unearthed a positive relationship between prenatal PFAS exposure and subsequent obesity [100]. Additionally, maternal PFAS exposure has been associated with specific DNA methylation alterations, with these PFAS-linked CpG sites mapping to gene regions pertinent to cardiovascular health and renal function [101]. These revelations prompt consideration of the potential linkage between PFAS exposure and other components of CKM syndrome, warranting further elucidation.

### 4.4. Polycyclic Aromatic Hydrocarbon

With their intrinsic characteristics, polycyclic aromatic hydrocarbons (PAHs) persist as pollutants, displaying a diverse array of biological toxicities [102]. PAHs emerge during the refining of coal, crude oil, and natural gas [102]. Human studies have found a relationship between PAH exposure and cardiovascular disease [81], metabolic syndrome [82], NAFLD [83], and kidney disease [67,103]. As PAHs can pass through the placental barrier, studies have shown that exposure to PAHs during pregnancy can result in developmental toxicity [104]. Benzo(a)pyrene (BaP), a major example of PAHs, has shown epigenetic actions, such as inhibiting the activity of DNA methyltransferases and increasing histone deacetylases (HDACs) [105]. Considering the crucial role of epigenetic regulation in developmental programming [106], the interplay between PAH exposure and epigenetic regulation behind CKM programming deserves further evaluation.

### 4.5. Air Pollution

Air pollution, one of the greatest threats to global health, is also a risk factor for CKM syndrome. Airborne pollutants, encompassing carbon monoxide (CO), ozone (O_3_), nitrogen oxides (NOx), sulfur dioxide (SO_2_), volatile organic compounds (VOCs), and respirable particulate matter, exhibit variances in their chemical compositions [107]. Particulate matter is generally categorized by its mean aerodynamic diameter as PM10 (<10 μm in diameter), PM_2.5_ (<2.5 μm), or ultrafine particles (UFPs, <0.1 μm). PM_2.5_ and PM_10_ are frequently studied particulate matter indices, and both have been linked to various components of CKM syndrome, including cardiovascular disease [84,108], diabetes [85], NAFLD [86], kidney disease [67,109], and hypertension [108]. Highlighted in certain observational studies within exposed populations is the correlation between maternal exposure to PM_2.5_ and negative outcomes in offspring, notably hypertension [110] and diabetes [111].

### 4.6. Heavy Metals

Considered the most significant threat to human health among all forms of pollution in drinking water and food are heavy metals, owing to their persistence in the environment and their bioavailability [112]. Epidemiological data indicate that chronic exposure to heavy metals, including cadmium (Cd), mercury (Hg), and lead (Pb), escalates the risks of cardiovascular disease [87], kidney disease [88], obesity [89], diabetes [89], and hypertension [89]. A meta-analysis comprising 13 studies demonstrates substantial links between Cd, Hg, Pb, and arsenic exposure during pregnancy and heightened risks of specific congenital heart diseases in offspring [113]. Additionally, a study revealed that elevated selenium levels are associated with an increased risk of congenital anomalies of the kidney and urinary tract (CAKUT) [114]. Reported in another investigation is an inverse relationship between maternal blood lead levels and kidney function in children aged 8–12 years who are overweight [115]. Furthermore, a study examining mother–infant pairs evaluates the impact of antenatal heavy metal exposures on childhood BP [115]. Although Cd shows no association with systolic BP overall, the inverse correlation between manganese and systolic BP is more pronounced at higher Cd levels [116].

## 5. Evidence from Animal Models: The Role of AHR in CKM Programming

While epidemiological observations suggest a correlation between environmental chemical exposures and CKM syndrome, there remains a scarcity of comprehensive information regarding antenatal chemical exposure and the manifestation of CKM syndrome in adulthood. It is important to note that these observational studies alone cannot definitively establish a causal relationship between antenatal chemical exposure and adult CKM syndrome. Moreover, these human studies fail to elucidate the molecular mechanisms underlying the development of CKM syndrome or provide strategies for reprogramming.

To delve into the role of the AHR in antenatal chemical exposure-induced CKM programming, animal models serve as invaluable tools. They facilitate the understanding of mechanisms and aid in the development of preventive strategies. Table 2 outlines animal studies that demonstrate the association between maternal chemical exposure and subsequent CKM syndrome in offspring, with a particular focus on AHR signaling. This review exclusively focuses on chemical exposures occurring during pregnancy and/or lactation, with an emphasis on reporting offspring outcomes commencing from childhood onwards.

Table 2 illustrates that rodents are the predominant animal species utilized, with large animals not currently employed for studying similar exposures. The programming effects of environmental chemicals have been documented in rats aged between 7 and 21 weeks, corresponding to human ages from childhood to young adulthood [142].

The earliest AHR agonists identified were typically constituents of environmental chemicals including dioxins, BPA, phthalates, and PFOS, as well as polycyclic aromatic hydrocarbons [143]. Various chemicals have been assessed, including TCDD [117,118,119,120,121], BPA [122,123,124,125,126], DEHP [127,128,129], DBP [130,131], perfluorooctane sulfonic acid (PFOS) [132], BaP [133], Cd [134,135,136], and PM_2.5_ [137,141]. Maternal exposure to the AHR ligand TCDD induces hypertension in offspring, correlated with AHR/CYP1A1 induction and TH17-mediated renal inflammation [118]. Additionally, cardiovascular dysfunction and kidney malformations have been observed in rat offspring prenatally exposed to TCDD [121].

Similarly to TCDD, BPA acts as an AHR ligand [35]. Exposure during pregnancy and lactation induces various components of CKM syndrome in rats, including kidney disease, obesity, NAFLD, hypertension, insulin resistance, and hyperlipidemia [122,123,124,125,126]. In a rat model of maternal BPA exposure, adult offspring developed hypertension alongside increased protein levels of AHR and mRNA expression of AHRR, CYP1A1, and ARNT in the offspring kidneys [125].

DEHP and DBP, two widely used phthalates acting as endocrine disruptors and AHR ligands, exhibit detrimental effects on offspring when maternally exposed. These effects include kidney dysfunction, hypertension, abnormal adipogenesis, and glucose metabolism alterations [127,128,129,130,131]. Maternal exposure to DBP is also implicated in offspring exhibiting kidney dysfunction, renal fibrosis, and obesity [130,131].

PFOS, another investigated environmental chemical, induces hypertension in both male and female rat offspring at 16 weeks of age when the dams are exposed during gestation [132]. Despite being known to activate AHR, PFOS’s mechanism in this study remains unclear [144]. BaP, a polycyclic aromatic hydrocarbon, contributes to cardiovascular disease via AHR activation [145], and gestational exposure leads to offspring hypertension [133].

Maternal heavy metal exposure studies indicate Cd is the primary cause of adverse cardiovascular–kidney–metabolic outcomes programmed by early life exposure [134,135,136]. Prenatal Cd exposure in rats leads to kidney disease features in some studies, and obesity, hyperlipidemia, insulin resistance, and steatosis in a sex-specific manner [134,135,136].

Moreover, prenatal exposure to PM_2.5_ has been linked to hypertension, cardiac hypertrophy, obesity, glucose intolerance, and pancreatic islet dysfunction in rodents [137,138,139,140,141]. Notably, AHR is implicated in PM_2.5_′s prooxidative and pro-inflammatory effects [146].

## 6. Reprogramming Strategies Targeting AHR Signaling

In CKM programming, the pivotal roles played by the AHR underscore its significance as a potential therapeutic target. Indeed, the pharmacotherapy of numerous diseases has explored the targeting of the AHR, as extensively reviewed elsewhere [25,147,148,149]. Functioning as a ligand-driven receptor, the AHR exhibits complex pharmacology wherein its activation is contingent upon the type and concentration of ligands [147]. Ligands for the AHR can be categorized into three distinct groups: full agonists, partial agonists, and antagonists. Both agonists and antagonists bind to the receptor, yet only agonists induce a response. Partial agonists, on the other hand, stimulate a sub-maximal response despite occupying all the receptor sites; when paired with a full agonist, partial agonists act as functional antagonists [150]. Moreover, the activity of the AHR can be modulated by mechanisms independent of ligand binding [148].

Significantly, AHR facilitates developmental programming not only during gestation but also in the early stages of postnatal ontogenesis. This is crucial as interventions targeting AHR can be employed as reprogramming strategies during both pregnancy and the early postnatal period. Presently, various compounds that interact with the AHR have been identified as potential interventions for reprogramming to prevent CKM syndrome, including tryptophan metabolites, resveratrol, and butyrate. Each of these will be discussed sequentially.

### 6.1. Tryptophan Metabolites

Tryptophan undergoes conversion into a series of metabolites, many of which have been identified as ligands for the AHR (e.g., indole and tryptamine) [143]. In the gut, tryptophan metabolism traverses three principal pathways, encompassing the kynurenine pathway, the indole pathway, and the serotonin pathway [151]. Within a maternal CKD-induced hypertension model, the therapeutic efficacy of tryptophan in lowering BP is associated with its modulation of the AHR signaling pathway [152].

So far, the focus of research on microbial-derived metabolites involved in AHR modulation has primarily centered on tryptophan metabolites. While numerous microbial-derived tryptophan metabolites have demonstrated the ability to bind to and regulate AHR activity, only a few have been thoroughly investigated for their potential to reprogram AHR-related inflammatory responses and mitigate CKM syndrome. Nonetheless, additional studies are warranted to further elucidate their mechanisms and potential therapeutic applications.

Kynurenine, an AHR ligand, emerges through the degradation of tryptophan catalyzed by the enzyme indoleamine 2,3-dioxygenase (IDO). In the context of CKD, elevated kynurenine levels indicate activation of the IDO–kynurenine pathway [153]. IDO exhibits the capacity to foster the differentiation of Treg cells while impeding the differentiation of TH17 cells. TH17 cells, known for their production of interleukin 17 (IL-17), play a role in inflammation and tissue damage. Given kynurenine’s potential to incite AHR-mediated inflammation, inhibitors of IDO present themselves as promising targets for the treatment of cardiovascular disease [154]. Considering the regulatory influence of AHR on both Treg and TH17 cells [52], and the participation of several microbial tryptophan catabolites as AHR ligands in the developmental programming of kidney disease and hypertension [155], further investigation is warranted to comprehensively understand the protective role of tryptophan metabolites and IDO inhibitors in modulating CKM syndrome of developmental origin.

### 6.2. Resveratrol

Studied for their AHR modulatory potential, polyphenols have garnered attention as compounds capable of reaching cells and potentially influencing AHR activity across the gut and other organs [149]. Among these polyphenols, several have been identified as AHR ligands, exhibiting either agonistic or antagonistic properties [143]. Despite numerous published studies highlighting the anti-inflammatory effects of various polyphenol types in the prevention and treatment of diverse diseases [156], investigations into the beneficial actions of resveratrol specifically in AHR-related inflammation in various animal models of CKM programming remain limited.

Resveratrol, a natural polyphenol abundant in grapes, is renowned for its antioxidant, anti-inflammatory, and prebiotic properties, and its ability to modulate AHR [157]. Resveratrol has been characterized as an antagonist of the AHR, capable of inhibiting the activation of members of the CYP1 family by impeding the recruitment of the transcription factors AHR and ARNT to XREs within the enhancer regions of CYP1 family genes [158,159].

It has been proposed as a reprogramming strategy to forestall cardiovascular disease, kidney disease, and metabolic syndrome [160,161]. Previous research has shown that TCDD-induced hypertension correlates with AHR activation and TH17-induced renal inflammation [118]. Conversely, supplementation with resveratrol during gestation and lactation can counteract TCDD-induced AHR signaling activation and TH17 responses. Similarly, perinatal resveratrol therapy restores maternal BPA exposure-induced increases in AHR protein levels and mRNA expression of AHRR, CYPA1A1, and ARNT [125]. Furthermore, resveratrol has been documented to function as an antagonist of the AHR, showing efficacy in mitigating offspring hypertension in alternative models of the developmental origins of hypertension [120,162].

Despite its advantages, the challenge of translating basic scientific findings into clinical practice is posed by the limited bioavailability of resveratrol [163]. To address this hurdle, previous efforts have centered on esterifying resveratrol with butyrate, thereby producing resveratrol butyrate esters (RBEs) with the aim of enhancing efficacy [164]. An improvement in hyperlipidemia and obesity in female progeny and hepatic steatosis in male offspring, both induced by maternal exposure to BPA, have been demonstrated in studies through the administration of low-dose RBEs (30 mg/L) [123,124]. In a maternal DEHP exposure model, low-dose RBE treatment significantly shielded adult rat offspring against hypertension, accompanied by a reduction in renal mRNA expression of CYP1A1 and ARNT [128].

### 6.3. Butyrate

Butyrate, a prevalent short-chain fatty acid (SCFA) derived from gut microbiota, exerts its effects through various mechanisms, including acting as a histone deacetylase (HDAC) inhibitor, signaling via SCFA receptors, or functioning as a postbiotic [165,166]. Reports indicate that butyrate can activate the AHR and enhance the functions of AHR activated by ligands [148]. While butyrate itself does not directly bind to AHR, it can induce the nuclear translocation of AHR and activate AHR independently of its HDAC activity and SCFA receptors [167]. Given that perinatal supplementation with butyrate has demonstrated protective effects against offspring hypertension and metabolic dysfunction in various developmental programming models [168,169,170], further investigation is warranted to elucidate the potential role of the AHR signaling pathway in these protective actions.

### 6.4. Others

AHR is a pivotal player in orchestrating epigenetic regulations impacting transcriptome alterations, chromatin architecture adjustments, and involvement in crucial signaling pathways [171]. Epigenetic mechanisms such as aberrant DNA methylation, histone modification, and microRNAs can drive changes in gene expression, potentially leading to developmental programming [172]. Chemical exposure-related developmental programming implicates various molecular pathways including oxidative stress, dysregulated nutrient-sensing signals, aberrant RAS, reduced nephron numbers, and dysbiotic gut microbiota, all intertwined with epigenetic programming [172,173,174,175,176,177]. Given the interconnectedness of these mechanisms with AHR signaling, there exists significant potential for cross-talk among them in the context of CKM programming. Consequently, targeting AHR-related epigenetic modifications within these pathways could offer promising avenues for intervention.

Despite the identification of numerous AHR ligands across diverse chemical structural classes [143,147,148], only a subset of them has been scrutinized for their effects on developmental programming. Therefore, further investigation is warranted to comprehensively understand the impact of AHR agonists/antagonists on key molecular pathways. This deeper understanding is essential for developing AHR-targeted reprogramming therapies aimed at mitigating CKM syndrome.

Furthermore, previous research has indicated that genetic variability in response to AHR ligands may highlight specific genes that could be under the regulation of AHR [178,179]. Identifying these genes has the potential to enhance comprehension of the AHR’s involvement in developmental programming and could potentially pave the way for therapeutic interventions targeting adverse AHR-mediated CKM phenotypes.

## 7. Conclusions and Perspectives

**Figure 3:**
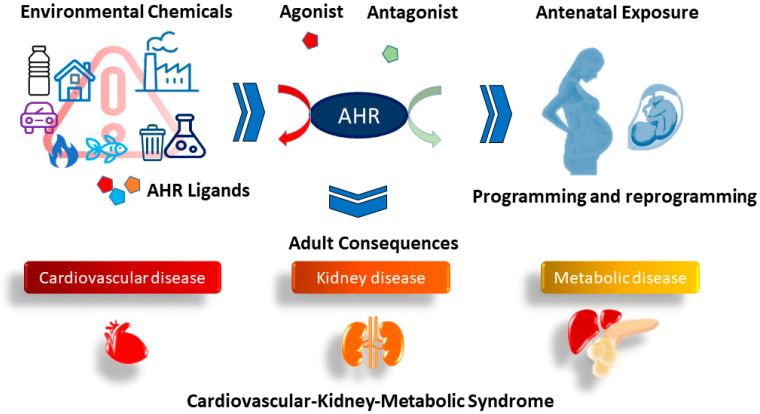
The AHR connects antenatal chemical exposure to CKM programming and reprogramming.

The reviewed literature collectively suggests the impact of early developmental exposure to environmental chemicals on offspring’s cardiovascular–kidney–metabolic health, culminating in CKM syndrome. The emerging concept from animal studies posits the intimate involvement of the AHR signaling pathway in CKM programming, induced by antenatal chemical exposure. Despite indications from animal models proposing that early interventions targeting AHR modulation could avert CKM syndrome, there persists a scarcity of human trials exploring their clinical translation. The relationship between antenatal chemical exposure and CKM programming and reprogramming via AHR links is depicted in Figure 3.

The impact of chemical exposure across various developmental stages can exhibit notable variations. Exposure during the initial phases of fetal development has the potential to interfere with organogenesis, while exposure later in development could impact the growth and functionality of organs that have already formed. By taking into account the timing of exposure and the vulnerability of organs, distinct effects on CKM programming can arise. Further exploration through animal studies is imperative to gain deeper insights into how exposure to different chemicals at various developmental stages can contribute to specific components of CKM syndrome later in life and to what extent. Another unresolved aspect pertains to the predominant use of mother–child cohorts in epidemiological studies, which pose challenges in extending observations into adulthood. There is a pressing need for additional long-term follow-up studies to comprehensively elucidate the chronic effects of antenatal chemical exposure mediated through AHR.

There exists an urgent imperative for multidisciplinary endeavors to undertake investigations discerning hazardous chemicals within the environment. Throughout pregnancy and early childhood, it is crucial to prioritize avoiding exposure to harmful substances and toxins in various settings such as the home, workplace, and recreational activities, as this is essential for promoting cardiovascular–kidney–metabolic well-being. While numerous environmental chemicals have been identified thus far, proactive efforts should persist in uncovering additional potentially harmful chemicals.

The growing spectrum of individual compounds that bind to and influence AHR-mediated responses and genes is continuously broadening [35,180,181]. This includes a wide range of structurally diverse synthetic chemicals, microbial metabolites, phytochemicals, and endogenous biochemicals. The potential exists within some of these compounds to serve as selective modulators of AHR for reprogramming strategies [147]. However, a significant caveat in the development of selective AHR modulators is the inability to readily predict their response selectivity as agonists or antagonists. The double-edged sword effects of AHR necessitate comprehensive testing to identify the optimal ligand for specific clinical applications.

In conclusion, antenatal exposure to environmental chemicals serves as a significant pathogenetic link in the developmental programming of CKM syndrome. With an enhanced comprehension of AHR’s role in CKM programming, AHR modulators show promise in maximizing benefits without exacerbating toxicity, thereby averting maternal chemical-induced CKM syndrome.

## Figures and Tables

**Table 1:** Major sources of environmental chemicals and their associated CKM syndrome in human studies.

Environmental Chemicals	Common Substances or Derivatives	Major Sources	Exposure-Associated CKM Phenotypes
Dioxins	TCDD, PCDF, PCDD, PCB	Consumption of animal products rich in fat, pesticide production, wood pulp bleaching, and the process of waste incineration	Cardiovascular disease [63], diabetes [64], metabolic syndrome [65], hypertension, and kidney disease [66,67]
Bisphenol A	BPA	Plastic containers, lenses, medical tubing, and apparatus	Cardiovascular disease [68], diabetes [69], obesity [69], NAFLD [70], hypertension, and kidney disease [66,67]
Phthalates	DEHP, DBP	Vinyl plastics, cosmetics, shampoos, medical devices, and food packaging	Cardiovascular disease [71], diabetes [72], metabolic syndrome [73], NAFLD [74], hypertension [67], and kidney disease [66,67]
Organophosphate flame retardants	DPHP, TPHP, TDCPP	Plastics, rubbers, textiles, upholstered furniture, building materials, and electronic equipment	Cardiovascular disease [75], metabolic syndrome [76], kidney disease [77], and hypertension [78]
Microplastics	MPs	Various foods, including drinking water, seafood, milk, sugar, and salt	Cardiovascular disease [79]
Per- and polyfluoroalkyl substances	PFOA, PFOS	Firefighting foams, non-stick cookware, stain-resistant fabrics, water-repellent coatings, and food packaging materials	Obesity, diabetes, NAFLD [80], hypertension, and kidney disease [66,67]
Polycyclic aromatic hydrocarbon	BaP	Vehicle exhaust, industrial processes, tobacco smoke, and grilled food	Cardiovascular disease [81], metabolic syndrome [82], NAFLD [83], and kidney disease [66]
Air pollution	PM_10_, PM_2.5_	Factories and manufacturing plants, transportation, agriculture, and waste management	Cardiovascular disease [84], diabetes [85], NAFLD [86], hypertension [67], and kidney disease [67]
Heavy metals	Pb, Cd, Hg	Manufacturing processes, emissions from vehicles, combustion of fossil fuels, improper disposal of electronic waste and batteries, and contaminating soil, water, and air	Cardiovascular disease [87], kidney disease [88], obesity [89], diabetes [89], and hypertension [89].

TCDD, 2,3,7,8-tetrachlorodibenzo-p-dioxin; PCDF, polychlorinated dibenzo-p-furan; PCDD, polychlorinated dibenzo-p-dioxin; PCB, dioxin-like polychlorinated biphenyl; BPA, bisphenol A; DEHP, di-2-ethylhexylphthalate; DBP, di-n-butyl phthalate; DPHP, diphenyl phosphate; TPHP, triphenyl phosphate; TDCPP, Tris-(1,3-dichloroisopropyl)phosphate; MPs, microplastics; PFOS, perfluorooctane sulfonic acid; PFOA, perfluorooctanoic acid; BaP, benzo(a)pyrene; PM_10_ (particulate matter < 10 mm in diameter), PM_2.5_ (particulate matter < 2.5 mm); Pb, lead; Cd, cadmium; Hg, mercury.

**Table 2:** Overview of animal models of antenatal chemical-induced programmed CKM syndrome related to AHR signaling.

Chemical	Exposure Dose and Period	Species	Age at Evaluation (Weeks)	CKM Phenotypes	Refs.
TCDD	200 ng/kg orally on gestational days 14 and 21 and days 7 and 14 after birth	SD rats/M	12	Hypertension	[117]
TCDD	200 ng/kg in four once-weekly oral doses throughout pregnancy and lactation	SD rats/M	12	Hypertension	[118,119]
TCDD	200 ng/kg in four weekly oral doses throughout pregnancy and lactation	SD rats/M	16	Hypertension	[120]
TCDD	6.0 µg/g orally on gestational day 14.5	C57BL/6N mice/M	12	Cardiovascular dysfunction and kidney malformation	[121]
BPA	10 or 100 mg/kg/day throughout gestational days 9–16	OF1 mice/ M and F	5	Kidney dysfunction	[122]
BPA	50 μg/kg/day throughout gestation and lactation	SD rats/F	7	Obesity	[123]
BPA	50 μg/kg/day throughout gestation and lactation	SD rats/F	7	NAFLD	[124]
BPA	50 mg/kg/day throughout gestation and lactation	SD rats/M	16	Hypertension	[125]
BPA	10 or 100 μg/kg/day throughout gestational days 9–16	SD rats/F	16	Insulin resistance and hyperlipidemia	[126]
DEHP	0.25 or 6.25 mg/kg/day throughout pregnancy	Wistar rats/ M and F	21	Kidney dysfunction and hypertension	[127]
DEHP	10 mg/kg/day throughout pregnancy and lactation	SD rats/M	12	Hypertension	[128]
DEHP	0.2, 2, or 20 mg/kg/day throughout pregnancy and lactation	ICR mice/M	12	Abnormal adipogenesis and glucose metabolism	[129]
DBP	850 mg/kg/day throughout gestational days 14–18	SD rats/M	8	Kidney dysfunction and renal fibrosis	[130]
DBP	33, 66, or 132 mg/kg/day from gestational day 7 throughout postnatal day 21	SD rats/F	12	Obesity	[131]
PFOS	50 μg/mL from gestational day 4 until delivery	SD rats/ M and F	16	Hypertension	[132]
BaP	600 or 1200 mg/kg/day throughout gestational days 14–17	LEH rats/ M and F	8	Hypertension	[133]
Cd	Cd chloride 2.0 or 2.5 mg/kg/day on gestational days 8, 10, 12, and 14	SD rats/M	7	Kidney injury	[134]
Cd	Cd chloride 0.5 mg/kg/day throughout pregnancy	Wistar rats/ M and F	8	Kidney dysfunction	[135]
Cd	500 ppb CdCl_2_ in drinking water throughout pregnancy to postnatal day 10	CD-1 mice/ M and F	17	Obesity, hyperlipidemia, insulin resistance, and steatosis in F	[136]
PM_2.5_	PM_2.5_ exposure for 16 weeks prior to delivery	C57BL/6N mice/M and F	12	Hypertension	[137]
PM_2.5_	PM_2.5_ exposure 300 µg/m^3^ for 2 h/day throughout	C57BL/6N mice/M and F	12	Cardiac hypertrophy	[138]
PM_2.5_	Oropharyngeal drip of PM_2.5_ (1.0 mg/kg) on gestational days 8, 10, and 12	SD rats/M	14	Hypertension	[139]
PM_2.5_	Concentrated ambient PM_2.5_ exposure throughout gestation and lactation	C57BL/6N mice/M and F	22	Obesity	[140]
PM_2.5_	Diesel exhaust PM_2.5_ 8.6 μg/day intratracheal instillation throughout pregnancy and lactation	C57BL/6N mice/M and F	22	Glucose intolerance and pancreatic islet dysfunction	[141]

TCDD, 2,3,7,8-tetrachlorodibenzo-p-dioxin; BPA, bisphenol A; DEHP, di-2-ethylhexylphthalate; DBP, di-n-butyl phthalate; PFOS, perfluorooctane sulfonic acid; BaP, benzo(a)pyrene; Cd, cadmium; PM_2.5_ (particulate matter < 2.5 mm); SD, Sprague–Dawley rat; LEH, Long–Evans Hooded; OF1, Oncins France 1; M, male; F, female.

## Data Availability

Data are contained within the article.
